# Supplementary material for: Differential Neuropathology, Genetics, and Transcriptomics in Two Kindred Cases with Alzheimer’s Disease and Lewy Body Dementia
Source: Biomedicines. 2022 Jul 13;10(7):1687. doi: 10.3390/biomedicines10071687 (PMC9313121; doi:10.3390/biomedicines10071687)
Supplement: Supplementary file 1 [file biomedicines-10-01687-s001.zip › Supplementary Data.pdf]

Table S1

Primary Antibodies	Host	Dilution	Pretreatment
Anti-βamiloid (4G8) (Biolegend)	Mouse (monoclonal)	1:1000	70% formic acid in H2O 10 minutes
Phospho-Tau (Ser202, Thr205) (AT8) (Thermo Scientific; MN1020)	Mouse (monoclonal)	1:200	
Alpha-Synuclein (KM51) (Novocastra; NCL-L-ASYN)	Mouse (monoclonal)	1:500	1) three steps in microwave for 2/1/2 minutes with citrate buffer 0,01 M pH 6;<br>2) 70% formic acid in H2O 10 minutes
Anti Phospho TDP43 (Ps409/410-2) (CosmoBio; TIP-PTD-P02)	Rabbit (polyclonal)	1:4000	Three steps in microwave for 2/1/2 minutes with citrate buffer 0,01 M pH 6
AntiNeuN (A60) (Chemicon; MAB377)	Mouse (monoclonal)	1:1000	Three steps in microwave for 2/1/2 minutes with citrate buffer 0,01 M pH 6
Glial Fibrillary Acidic Protein (Dako; Z0334)	Rabbit (polyclonal)	1:1000	
**Secondary Antibodies**	**Host**	**Dilution**	**Pretreatment**
EnVision+Sysyem+HRP (Dako 4001)	Anti-mouse	1:2	
EnVision+Sysyem+HRP (Dako 4003)	Anti-rabbit	1:2	

Table S1. Complete list of primary and secondary antibodies, their characteristics, dilutions and pretreatments

Table S2

Hereditary Hypothesis	Variants “in common”	
	**Variants in mother (het/homo)**	**Variants in son (het/homo)**
Autosomic Recessive	Homo	Homo
Autosomic Dominant	Het	Het
Compound Heterozygosity	Het + Het	Het + Het
X-Linked Recessive	Homo	Het
X-Linked Dominant	Het	Het
**Hereditary Hypothesis**	**Variants “not in common”**	
	**Variants in mother (het/ -)**	**Variants in son (het/homo/*de novo*)**
Autosomic Recessive	Het	Homo
Autosomic Dominant	-	*de novo*
Compound Heterozygosity	Het	Het + *de novo*
X-Linked Recessive	-	Homo
X-Linked Dominant	-	*de novo*

Table S2. List of the hereditary hypotheses and the possible combinations of how the variants “in common” must be present in mother and son to explain their clinical phenotypes and how the variants “not in common” must be present in mother and son to explain the clinical phenotype only of the son. **Het**: heterozygous; **Homo**: homozygous.

Figure S1

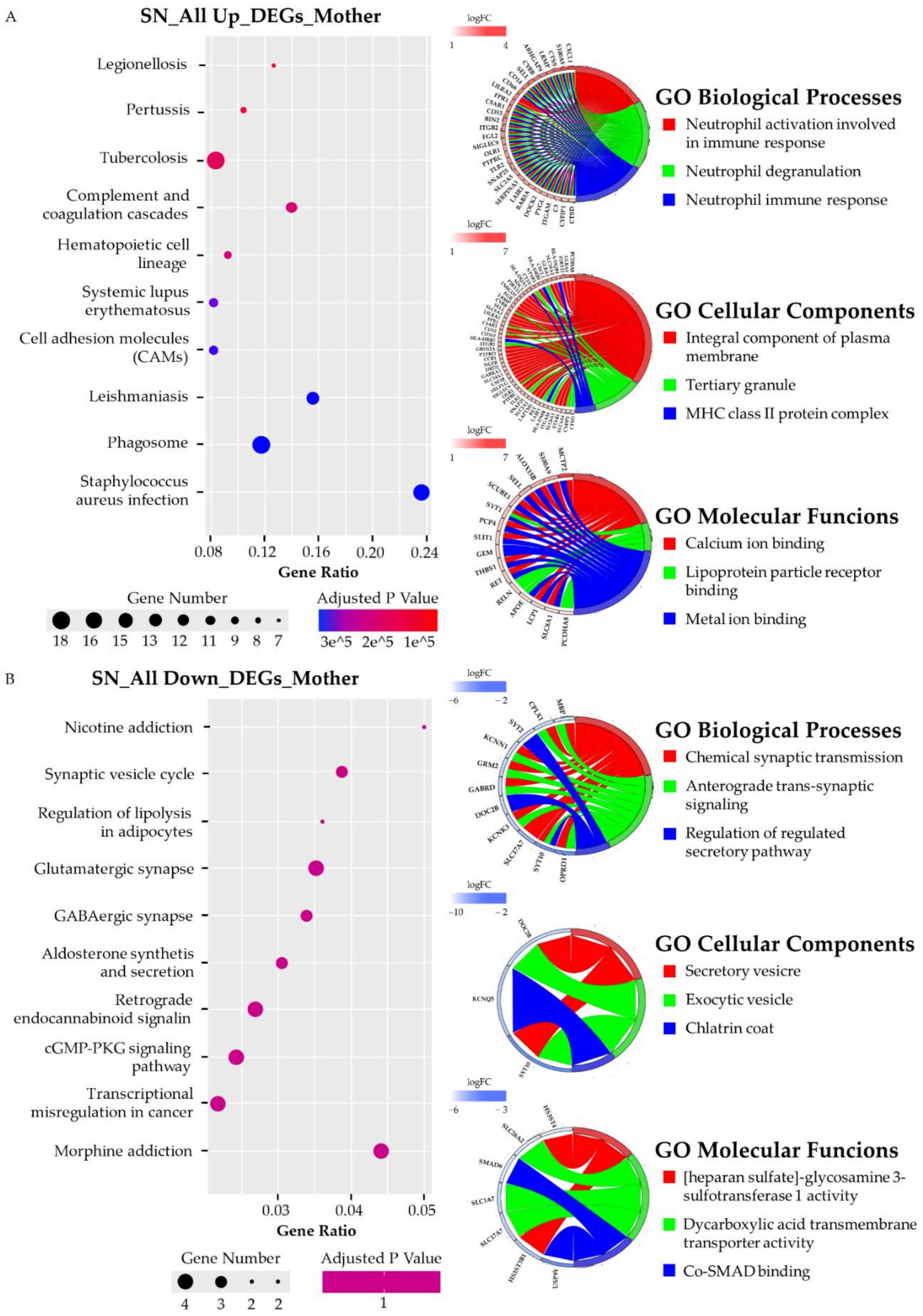

Figure S1. KEGG pathways and GOchord analysis relative to the DEGs of the Substantia Nigra of the mother (BB105). 1A) top ten KEGG pathways and top three GO enriched terms in terms of biological processes, cellular components and molecular functions related to the ALL UP DEGs; 1B) top ten KEGG pathways and top three GO enriched terms in terms of biological processes, cellular components and molecular functions related to the ALL DOWN DEGs.

Figure S2

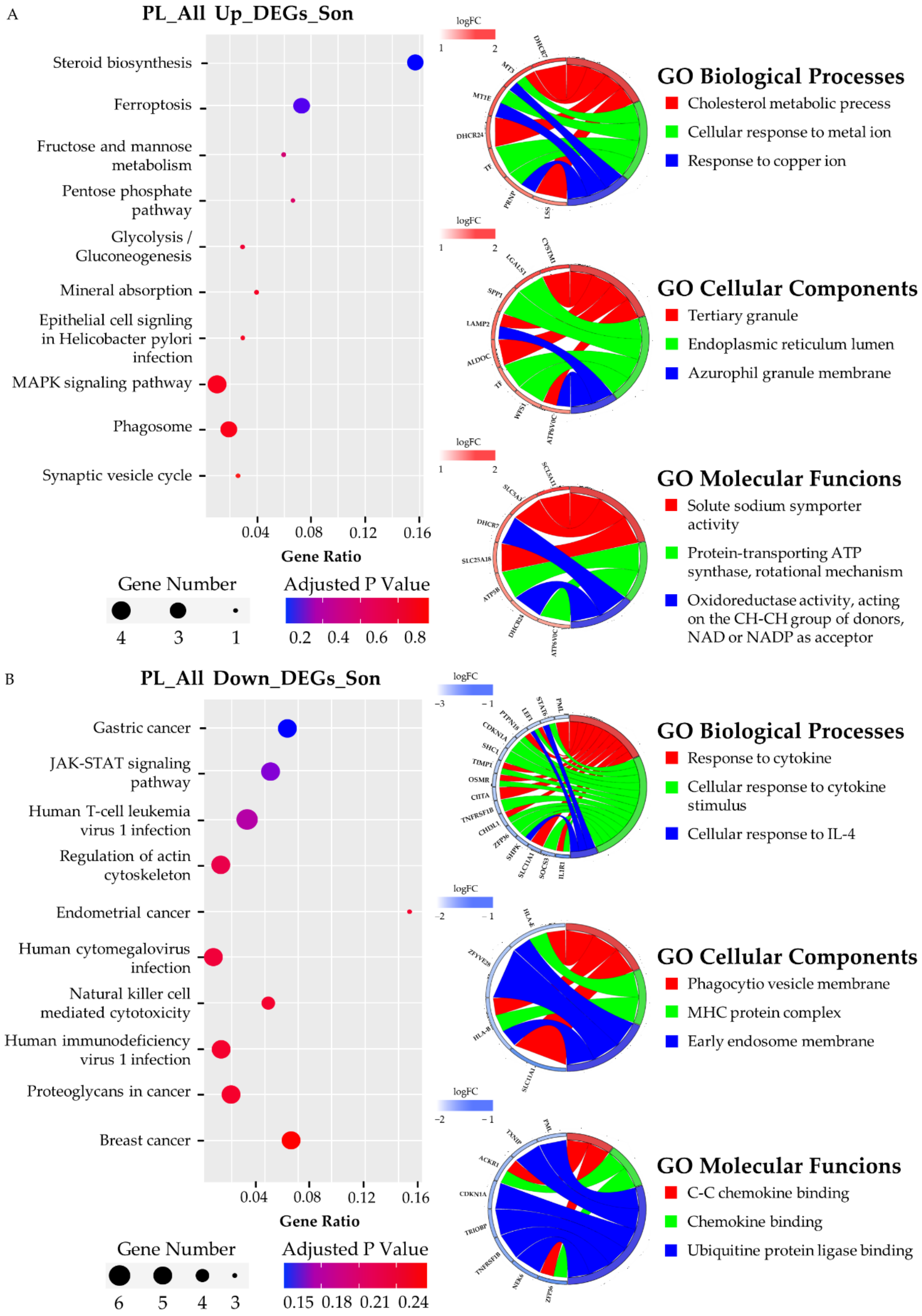

Figure S2. KEGG pathways and GOchord analysis relative to the DEGs of the Parietal lobe of the son (BB181). 2A) top ten KEGG pathways and top three GO enriched terms in terms of biological processes, cellular components and molecular functions related to the ALL UP DEGs; 2B) top ten KEGG pathways and top three GO enriched terms in terms of biological processes, cellular components and molecular functions related to the ALL DOWN DEGs.

Gene list (from top to bottom) of the GOchord "biological processes" relative to the SN_All Up_DEGs_SON: RIT2, SNAP25, GLRA1, NEFH, MCHR1, SLC17A6, CHRM2, BDNF, SYT1, HTR5A, SEMA3A, RAB3A, GPR149, KCNA1, NPY1R, NSG1, GABRG2, AMPH, SYN2, EPHA5, CHRM3, POU4F2, NPTX2, CNTN4, SYN1, SHH, RET, L1CAM, ROBO2, LINGO2, EPHA8, EFNA3, CXCR4, GRIK1, OPRD1, NAPB, LRRN1, CACNA1E, TUBB3, GAP43, GRM4, CDK5, CDH8, LGI1, LRFN5, DOC2A, OPRK1, KCNQ3, KCNK3, GABRA3, AFG3L2, DOK6, WNT5A, OPRL1, SCN1B, NELL2, SLC17A7, PTPRO, SSTR3, GABRA1, SLIT3, PAK3, CPLX1, MAP1B, GABRB2, GABRD, GRIN2B, EFNA5, CACNA1B, BSN, SYT5, GABBR2, DLGAP2, UNC5A, CHRNB2, SLC9A6, CACNB4, GLRB, GRIN2A, SLITRK3, ATL1, SEMA3C, SLC12A5, SPTB, CYFIP1, MAP1A, UNC13A, PPT1, CYFIP2, KIF3A, CPNE6, S100A6, SPTBN2, SPTAN1, GABRB3, GRM5, KCND5, PIK3CD, NPTX1, RAP1GAP, CACNA1A, SPTBN4, APBA1, CDK5R1, NAPA, GRIA4, CACNB1, DLG4, LRRN2, NRXN1, NRCAM.

Gene list (from top to bottom) of the GOchord "cellular components" relative to the SN_All Up_DEGs_SON: PCDHA8, KCNK4, GPR22, CRTAM, KCNK5, KCNA6, TNFRSF11B, VSTM5, NEFM, SLCO5A1, GLRA1, NEFH, KCNV1, MCHR1, TUSC3, CD8B, P2RY12, HLA-DQA1, SLC26A8, KCNC2, CHRM, MME, KCNA4, BDNF, HTR5A, ATP1A3, NEFL, KCNQ5, LRMP, SEMA3A, RAB3A, GPR149, IL12RB2, KCNK9, KCNA1, QRFPR, NPY1R, GABRG2, FLT3, HCN1, EPHA5, CD180, GRM8, PCDHA11, CHRM3, OLR1, TMIGD3, TPBG, GPR34, CNTN4, DNM1, SLC35A1, IL17RE, BDKRB2, P2RY13, CD83, SLC8A1, CD53, CYBB, CSF2RA, L1CAM, ROBO2, PAK1, SCN8A, CDK5, SCN1B, KCNC1, MAP1B, EPB41L3, KCNC3, ACTAY, KCNAB2, PPT1, MTMR2, CPNE6, GARS, STAT1, SPTBN4, CNTNAP2, SACS, CDK5R1, HSPA8, NRXN1.

Gene list (from top to bottom) of the GOchord "cellular components" relative to the SN_All Up_DEGs_SON: KCNK4, KCNK5, KCNA6, SNAP25, KCNV1, KCNC2, KCNA4, SYT1, KCNQ5, KCNK9, KCNA1, SYT2, NSF, HCN1, KCNS1, KCNH6, KCNH5, SYT15, GRIK1, NAPB, KCNS3, CACNA1E, SYT3, STXBP5L, CACNG8, VAMP1, CACNG2, KCNH7, SYT13, DOC2A, PRRT2, KCNQ3, KCNK3, KCNT1, STXBP1, CNGB1, SYT4, KCNA2, CPLX1, KCNC1, KCNK1, CPLX2, CACNA1B, SYT7, SYT5, KCNC3, CACNB4, HCN3, UNC13A, NAPG, KCNK12, CACNA1I, STXBP2, KCND2, STXBP2, CACNA1A, NAPA, CACNB1, KCNH2.
