# Supplementary figures and images for: Differential Neuropathology, Genetics, and Transcriptomics in Two Kindred Cases with Alzheimer’s Disease and Lewy Body Dementia
Source: Biomedicines. 2022 Jul 13;10(7):1687. doi: 10.3390/biomedicines10071687 (PMC9313121; doi:10.3390/biomedicines10071687)

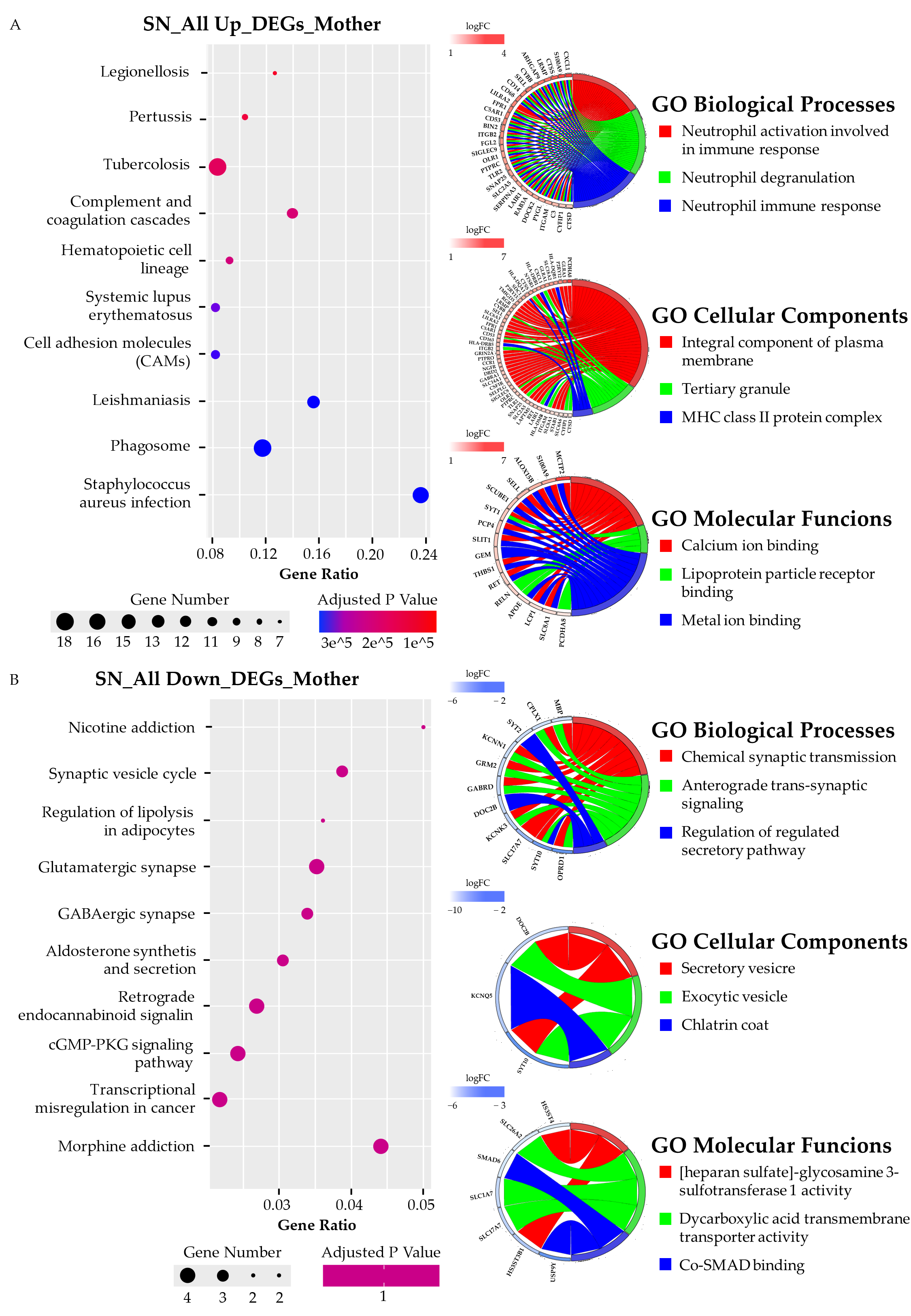

Supplement: Supplementary file 1 [file biomedicines-10-01687-s001.zip › Figure S1_300dpi.tif]

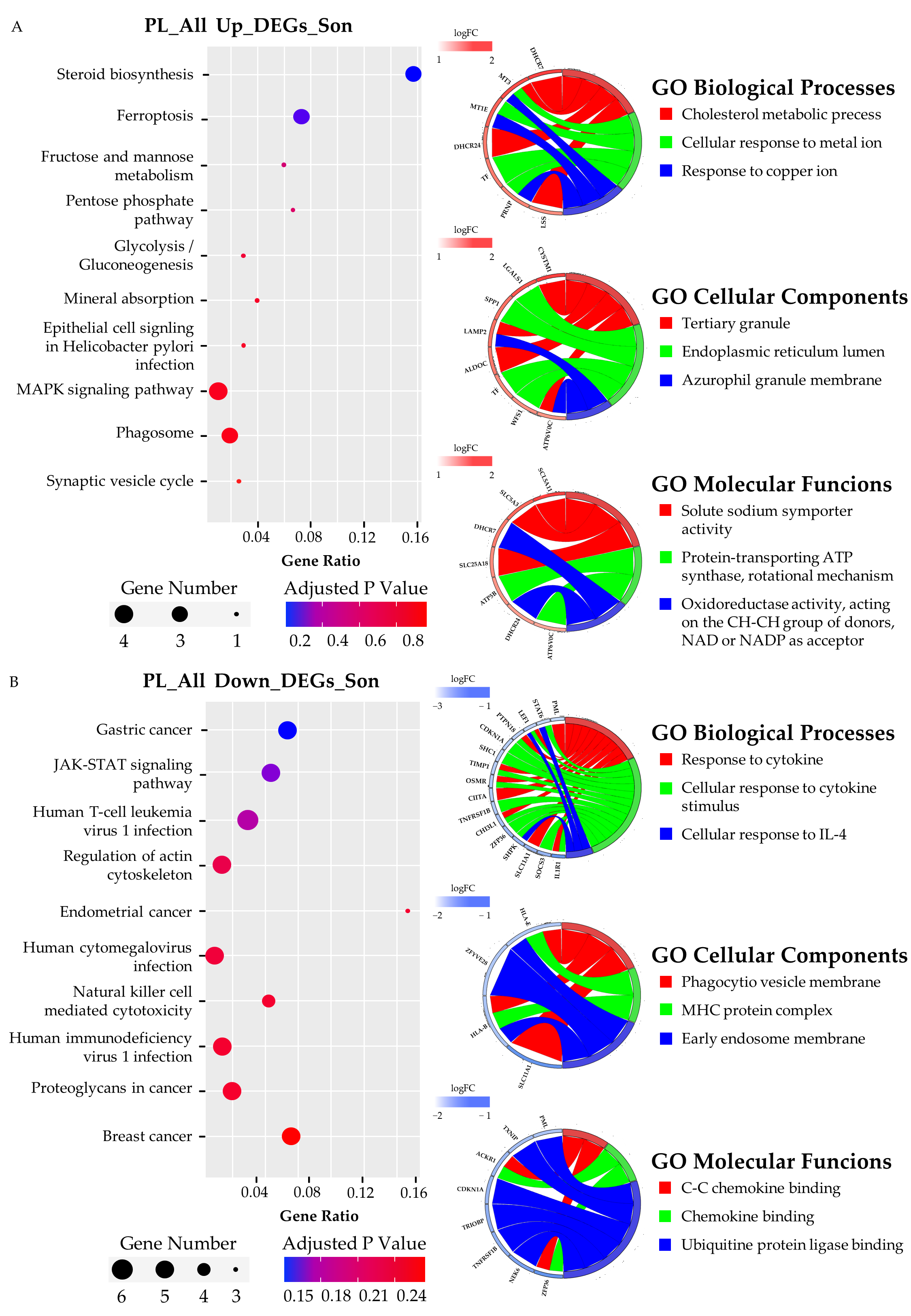

Supplement: Supplementary file 1 [file biomedicines-10-01687-s001.zip › Figure S2_300dpi.tif]
